# Lightweight Representation of Motion-Magnified Facial Dynamics for Micro Expression Sensing

**DOI:** 10.3390/s26123727

**Published:** 2026-06-11

**Authors:** Seungho Lee, Sangkon Lee

**Affiliations:** 1 School of Future Technology, Korea University of Technology and Education, Cheonan 31253, Republic of Korea; 2 School of Industrial Management, Korea University of Technology and Education, Cheonan 31253, Republic of Korea; sklee@koreatech.ac.kr

**Keywords:** micro expression recognition (MER), emotion recognition, motion magnification, micro expression sensing

## Abstract

Reliable monitoring of spontaneous affect is essential in biomedical sensing, where involuntary facial signals serve as objective indicators of physiological states. Micro expression recognition (MER) is particularly challenging due to the sub-second, low amplitude nature of these signals. Many existing MER methods rely on apex (peak) frame detection, making them sensitive to temporal localization errors and difficult to deploy in unconstrained settings. To address this, we propose an apex-free framework that analyzes facial dynamics by structuring motion-magnified features along a newly introduced magnification intensity axis. By applying Eulerian motion magnification across multiple discrete levels and collapsing the sequences into single accumulation images, we generate a multi-level representation of subtle facial dynamics without requiring frame-level annotations. A lightweight shared temporal mixer (STM) is employed to analyze the dynamic evolution of motion across the magnification intensity axis. Subsequently, a dual-branch convolutional neural network (CNN), processing low- and high-amplification regimes respectively, integrates a convolutional block attention module (CBAM) to capture subtle facial motion while effectively filtering out irrelevant noise. Our model is highly efficient, requiring only 0.94 M parameters and 262 MFLOPs, which is significantly lower than the computational demands of standard backbones such as ResNet18 or VGG16. To ensure the model generalizes to new individuals, we evaluated it by testing on subjects whose data was entirely excluded from the training process. Under this rigorous setup, the proposed method achieves approximately 80% and 70% accuracy on the CASME II and SMIC datasets respectively, showing performance comparable to, or in some cases, slightly above current state-of-the-art methods. Considering both the competitive accuracy and high computational efficiency, the proposed framework holds significant potential for practical integration into real-time affect monitoring systems, particularly within biomedical applications.

## 1. Introduction

Facial expressions are essential for emotional communication, and their automated analysis is widely utilized. Among these, micro expressions are uniquely valuable as they reveal involuntary and concealed emotions. Micro expression analysis has demonstrated practical utility in clinical assessment, deception detection, and human–computer interaction [1,2]. The growing demand for real-time deployment requires the development of robust and computationally efficient micro expression recognition (MER) methods.

Micro expressions are spontaneous, low-intensity facial movements with a duration of 1/25–1/3 s [3,4]. Their suppressed and transient nature makes automated recognition significantly more challenging than conventional facial expression recognition, as standard models struggle with data scarcity and low signal amplitude [5,6]. Prior research evolved from hand-crafted features like LBP-TOP [7,8] and optical flow [9] to deep learning models such as CNNs [10] and Transformers [11,12]. Recent deep learning methodologies have expanded to encompass advanced methodologies, including dual-branch attention mechanisms [13,14,15,16], direct learning of graph structures [17], regularization learning with action unit-guided augmentation [18], and fine-grained 3D facial reconstruction [19]. Additionally, motion magnification [20] has been frequently incorporated to enhance subtle deformations, improving the discriminability of these brief signals prior to feature extraction [21,22].

Despite this progress, two major limitations exist. First, many approaches rely heavily on apex frame detection, which involves identifying the single most expressive frame where the facial motion reaches its peak intensity. This creates a critical bottleneck because apex frame localization, whether manual or automated, is highly unreliable. Even minor inaccuracies in pinpointing this peak could significantly degrade recognition performance. For instance, even recent sophisticated networks in [13,16] remain inherently dependent on onset and apex frame pairs to construct temporal features, making them vulnerable when apex localization is less precise. Moreover, relying on such a singular snapshot discards vital temporal dynamics, compromising robustness in unconstrained environments where precise temporal annotation is unavailable. Second, computational efficiency remains a challenge. Current methods often employ high-capacity backbones, such as VGG16 [23] or deep ResNets [24], and complex attention mechanisms to compensate for small-scale data. These designs, often exceeding tens of gigaFLOPs and millions of parameters, are incompatible with real-time inference on edge devices. Furthermore, such high-capacity models risk overfitting on limited MER benchmarks, widening the gap between benchmark performance and practical deployment. While the latest research trends from 2023 to 2026 have attempted to partially address these challenges through diverse deep learning frameworks with a detection module to incorporate gender characteristics for MER [14], structural graph learning [17], regularized frameworks driven by action unit data augmentation [18], fine-grained 3D geometric facial reconstruction [19], and adaptive lightweight dual-stream designs like DSTNet [15], they still introduce substantial architectural complexity, heavy parameter overheads, or strict dependencies on external preprocessing. Consequently, a significant research gap exists in establishing a fundamentally lightweight, preprocessing-free framework that balances high representational capacity with extreme architectural compactness.

This paper introduces a new MER method based on a fundamentally different perspective on micro expression representation. Shifting away from apex frame dependency, our method leverages multi-level magnification of facial motion. This approach effectively scales subtle motion patterns across multiple intensities. Our key insight is that distinct magnification levels reveal complementary facial motion signals that are otherwise imperceptible. To this end, we generate *N* accumulation images by aggregating temporal differences, effectively encapsulating the cumulative motion patterns at each magnification intensity level. By treating these *N* images as a structured sequence along the magnification intensity axis, the proposed method simultaneously extracts dynamic temporal information and spatial features. The main advantage is that this *N*-dimensional representation is maintained throughout the network, ensuring multi-scale richness without sacrificing architectural efficiency.

To translate these multi-level representations into practical use, we propose an ultra-lightweight architecture specifically optimized to process a sequence of accumulation images. Central to our design, a temporal mixer (TM) [25] is seamlessly integrated to model the dynamic variations along the magnification intensity axis, while a convolutional block attention module (CBAM) [26] reinforces spatially discriminative facial features within a shallow convolutional neural network (CNN) backbone. Crucially, this structural synergy allows the model to capture rich multi-level information with minimal complexity, as these specific modules account for less than 0.5% of the total parameters and FLOPs. By effectively bridging the gap between high representational capacity and architectural compactness, our model achieves extreme efficiency with only 0.94 M parameters and 262 MFLOPs. Being 13× and 115× lighter than ResNet18 and VGG16 counterparts, the architecture could be suited for real-time inference on resource-constrained edge devices.

Experiments have been conducted on two widely used MER benchmark datasets, CASME II [27] and SMIC [7], following the standard three-class (Positive, Negative, and Surprise) protocol. Under the leave-one-subject-out (LOSO) cross-validation, the proposed method achieved accuracies of 79.31% on the CASME II and 68.29% on the SMIC. These results are comparable to or outperform existing state-of-the-art methods. The results further demonstrate that our framework is highly competitive, particularly in terms of computational efficiency.

The remainder of this paper is organized as follows. Section 2 reviews related work and analyzes its technical limitations. Section 3 details the proposed method, focusing on micro expression analysis with varying magnification intensities and the ultra-lightweight architecture design. Section 4 evaluates the proposed method against state-of-the-art approaches in terms of accuracy and efficiency. Finally, Section 5 concludes the paper.

## 2. Related Works

Early MER research has mostly relied on hand-crafted descriptors paired with traditional classifiers. Guided by Ekman and Friesen’s Facial Action Coding System (FACS) [28], researchers have developed various representations to quantify subtle muscle movements. Pfister et al. [8] have utilized LBP-TOP with temporal interpolation to capture spatiotemporal dynamics, inspiring numerous variants such as CLBP [29], FBLBP [30], and LBP-SIP [31]. In parallel, motion-based approaches have emerged, including STCLQP [32] and MDMO [9], which analyze patterns of magnitude and direction across facial regions of interest. While these features have been typically classified via a learned classifier (e.g., support vector machine (SVM) [8,9]) and have achieved baseline success, their discriminative power has often been limited by domain-specific assumptions and a struggle to fully capture the highly transient nature of micro expressions.

To better capture subtle micro-motions, researchers have increasingly utilized motion magnification as a preprocessing step. Eulerian Video Magnification (EVM), introduced by Wu et al. [20], has established a foundation by amplifying imperceptible color and motion variations. This has been subsequently adopted in MER to enhance muscle movement visibility: Oh et al. [21] have demonstrated that magnifying motion prior to feature extraction improves performance, while Li et al. [22] have integrated magnification into deep learning pipelines to boost discriminability. However, these methods typically operate at a single, fixed magnification level, creating a dilemma: insufficient magnification fails to make motions salient, while excessive levels introduce noise and distortion. Moreover, the dependency on apex frame detection has remained a bottleneck for robust deployment. These gaps motivate the need for a multi-level strategy that aggregates complementary cues across multiple magnification intensity levels without requiring precise apex localization.

The emergence of deep learning has driven a paradigm shift in MER, facilitating a transition from hand-crafted features to end-to-end spatiotemporal representations. Early deep learning approaches primarily utilized 2D CNNs to extract spatial features from keyframes or optical flow fields, which were subsequently combined with recurrent architectures like Long Short-Term Memory (LSTM) networks to capture temporal dynamics [33,34]. To jointly encode spatiotemporal relationships, 3D CNNs and Vision Transformers (ViTs) [11,12] have been introduced, demonstrating superior performance by modeling long-range dependencies and fine-grained facial interactions via self-attention mechanisms. To further maximize the discriminative power of deep networks on subtle facial changes, recent advanced architectures have evolved toward multi-stream feature fusion, attention-driven learning, and diverse geometric or structural optimizations:

(1) Multi-Stream Architectures: Multi-stream frameworks have been widely investigated to capture complementary facial dynamics. Khor et al. [13] have introduced a shallow dual-stream network designed to efficiently capture micro facial motion. Moving beyond single-task frameworks, Nie et al. [14] proposed GEME, a dual-stream multi-task learning network that incorporates auxiliary gender-based feature learning to enhance recognition accuracy. More recently, to address the computational burden of dual-stream systems, Liu et al. [15] have developed a lightweight dual-stream network enhanced by an adaptive strategy for efficient inference.
(2) Attention Mechanisms and Structural Learning: To focus on regional facial micro-movements, advanced attention and graph architectures have been deployed. Zhou et al. [16] have proposed Dual-ATME, a dual-branch attention network that integrates hand-crafted and automated attention region selection for MER. The Hand-crafted Attention Region Selection (HARS) branch explicitly crops the eyebrow and mouth regions as regions of interest (ROIs) based on AU statistical prior knowledge, while the Automated Attention Region Selection (AARS) branch employs an attention mechanism on the full-face input to automatically localize micro-expression-salient regions in a data-driven manner. Zhang et al. [17] have introduced a novel approach that achieves recognition based on the direct learning of graph structures, capturing topological facial relationships.
(3) Data Augmentation and 3D Reconstruction: To overcome the inherent data scarcity in MER, alternative structural modeling and augmentation paradigms have emerged. Zhou et al. [18] tackle cross-dataset variations by introducing a regularization learning framework coupled with Action Unit (AU)-[28,35] guided data augmentation. Furthermore, expanding MER into the 3D domain, Sun et al. [19] have explored fine-grained 3D facial reconstruction to capture deep geometric representations of subtle expressions.

Despite these significant performance gains, these high-capacity or multi-stream architectures (ranging from complex graph structures and 3D reconstructions to multi-branch networks) often impose substantial computational costs or rely heavily on precise preprocessing and domain-specific annotations such as AUs or gender labels. Moreover, such intricate designs could be susceptible to overfitting on limited MER data.

In contrast to the approaches discussed above, the proposed method offers several distinct advantages. First, it does not require apex frame localization, removing a significant technical bottleneck. Second, it leverages multi-level motion magnification, facilitating a comprehensive analysis of subtle facial dynamics from diverse intensity perspectives. Furthermore, as demonstrated by our experimental results, the ultra-lightweight architecture ensures exceptional training and inference efficiency. This combination of robust representation and architectural compactness positions our framework as a highly practical solution for real-world deployment.

## 3. Proposed Method

**Figure 1:**
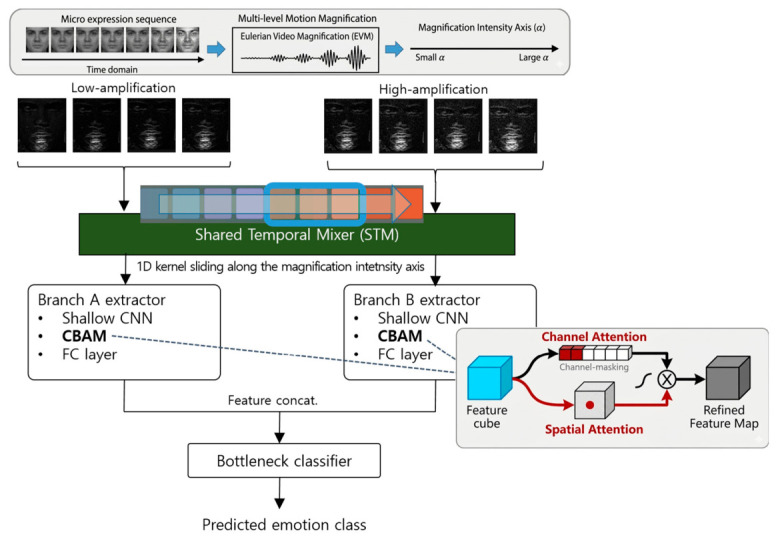
Overview of proposed method based on multi-level motion magnification.

The proposed method extracts subtle facial dynamics by transforming raw micro expression sequences into a multi-level representation of motion-magnified faces. For each of *N* discrete magnification factors α, the input sequence of facial expression images is processed using EVM to reveal subtle facial motion signals (refer to Figure 1). Each input sequence is considered to contain distinct temporal phases of a micro expression, specifically the onset, apex, and offset. To capture the motion-related information, we compute the accumulated pairwise frame differences within the sequence of magnified face images, which are then normalized into a single accumulation image. By iterating this across all *N* magnification intensities, we establish a distinct intensity-driven sequence that captures complementary motion patterns.

Figure 1 further illustrates the architecture for processing a sequence of *N* accumulation images. The accumulation images are partitioned into low-amplification and high-amplification regimes to capture distinct motion patterns. To model the continuous evolution of facial motion signals, both groups initially enter a shared temporal mixer (STM). This lightweight Conv3D module uses a 1D kernel along the magnification intensity axis to capture cross-level interactions that separate, independent designs would miss. Following the STM, the sequence is split into dedicated branch extractors. Each branch utilizes a shallow CNN, a CBAM for spatial refinement, and a fully connected (FC) layer to generate a compact feature vector. Finally, the concatenated features are processed by a bottleneck classifier for emotion class prediction (e.g., Positive, Negative, Surprise). This design balances multi-level dynamics and spatial appearance in an ultra-lightweight profile, suitable for data-scarce MER tasks.

The following subsections detail the sequence generation for the *N* accumulation images along the magnification intensity axis (Section 3.1) and the lightweight network designed for spatiotemporal analysis of the multi-level representation (Section 3.2).

### 3.1. Generation of Accumulation Image Sequence Along the Magnification Intensity Axis

**Figure 2:**
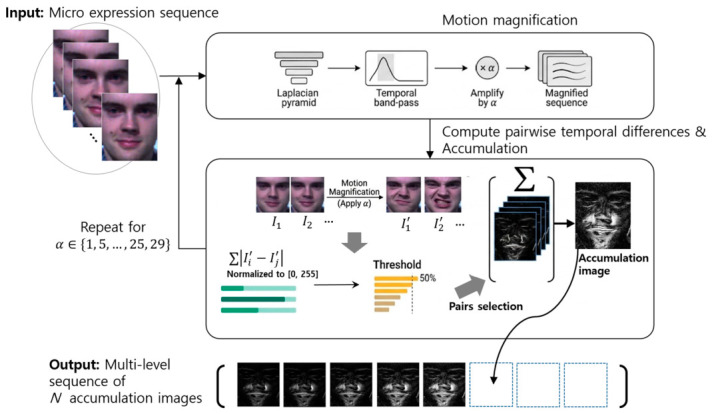
Illustration for obtaining the multi-level sequence of *N* accumulation images from a micro expression sequence.

**Figure 3:**
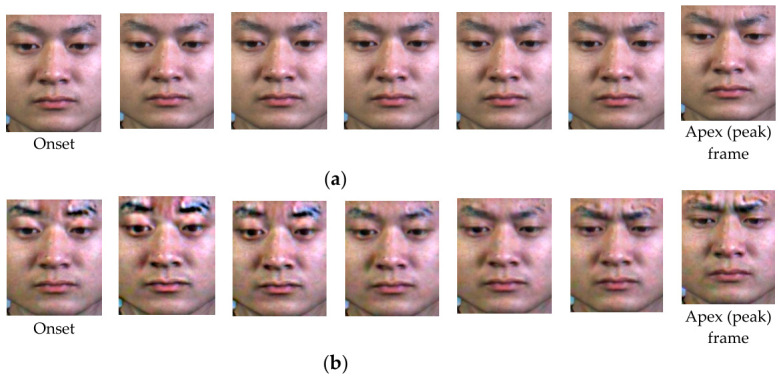
Comparison between (**a**) original and (**b**) magnified micro expression sequences sampled from the onset to the apex frame (α was set to 13 in this example). The motion magnification effectively emphasizes subtle facial motion otherwise invisible to the naked eye.

As illustrated in Figure 2, the generation of accumulation image sequence consists of: (1) independent EVM across *N* discrete magnification intensities α, and (2) threshold-based pairwise-difference accumulation. The effect of EVM is shown in Figure 3, which compares original micro expression frames from onset to apex with their magnified counterparts (α = 13). While the original frames appear nearly static, the magnified frames clearly reveal the underlying muscle contractions distinguishable. Such motion magnification is essential for MER, as these expressions involve extremely subtle and fleeting movements that often fall below the detection threshold of standard sensors and algorithms. By amplifying across multiple magnification intensities, the proposed method significantly enhances the signal-to-noise ratio (SNR) of facial motion signals, transforming them into a feature-rich representation suitable for robust emotion classification.

As shown in Figure 2, an input sequence of expression images $\left\{I_{1},I_{2},\;\ldots ,I_{m}\right\}$ is decomposed into a Laplacian pyramid to isolate spatial-frequency sub-bands. To extract micro-expression-specific signals, temporal filtering is applied to the sub-band coefficients, effectively filtering out static features and high-frequency noise. We set the frequency band to 1.0–15.0 Hz for EVM to specifically capture the rapid transient phases (onset and offset) of micro expressions. While the conventional range for heart-rate-based magnification is 0.5–3.0 Hz, micro expressions exhibit much higher temporal dynamics, often lasting as little as 1/15 to 1/25 of a second [3,4]. This frequency range ensures the suppression of low-frequency noise from global head movements (<1.0 Hz) while preserving the high-frequency muscular contractions essential for distinguishing subtle facial motion. The facial motion signals are then scaled by α to amplify the extracted micro-motion energy without affecting stationary spatial content. The subsequent reconstruction produces a magnified expression sequence $\left\{I_{1}^{\prime },I_{2}^{\prime },\;\ldots ,I_{m}^{\prime }\right\}$ yielding a sequence where subtle motion patterns are visibly emphasized without altering the underlying facial identity.

To condense the facial motion signal at a specific α into a single image, we compute absolute differences $D_{ij}={|\;I}_{i}^{\prime }-I_{j}^{\prime }\;|$ for all possible frame pairs (Figure 2). The pixel-wise temporal differences are derived from grayscale-converted frames instead of the original RGB color space. Each pair is assigned a scalar score $s_{ij}$ (spatial mean of $D_{ij}$*)* representing its motion energy. Pairs bracketing the expression peak yield high $s_{ij}$, while near-neutral pairs are dominated by noise. We retain only informative pairs by applying a 50% relative threshold (i.e., $s_{ij}\geq 0.5\times s_{max})$ and accumulating their difference images. The resulting image is min-max normalized to [0, 255]. In this way, we can effectively transform the temporal sequence into a cumulative spatial footprint, ensuring that the final representation focuses on salient muscle movement while suppressing uninformative temporal artifacts. It should be noted that, by decomposing an expression sequence into differences derived from independent frame pairs, the proposed method effectively captures facial dynamics while reducing unrelated information (such as facial identity) to expression change, as described in [35]. Crucially, this approach eliminates the need for apex frame localization, a common bottleneck in MER. Since discriminative features are aggregated from all valid pairs rather than a single apex frame, the resulting representation remains robust regardless of peak timing. This apex-free characteristic avoids the inaccuracies inherent in traditional localization steps, making it highly suitable for practical MER applications.

**Figure 4:**
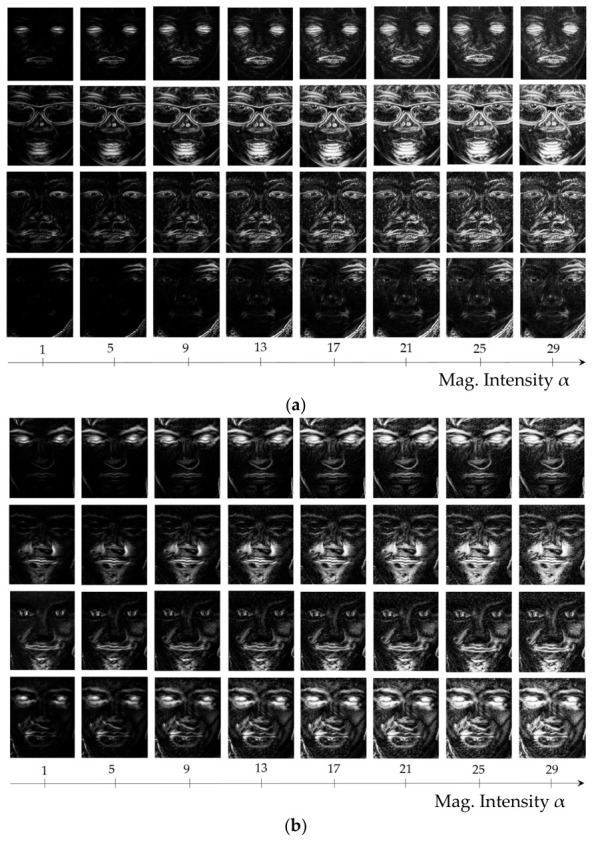
Examples of multi-level accumulation image sequences generated as a function of varying magnification intensities α. (**a**) CASME II dataset. (**b**) SMIC dataset.

The aforementioned stages are iterated for *N* magnification intensities, generating a sequence of accumulation images ordered by amplification strength as shown in Figure 4. Low α values produce sparse, high-fidelity images that capture only the most salient and localized muscle activations. As α increases along the magnification intensity axis, progressively subtle motion components are lifted above the noise floor, revealing secondary deformations that were previously imperceptible. However, this enrichment is accompanied by spatial saturation and spreading in the primary motion regions, particularly evident at higher intensities (e.g., α = 25, 29). Consequently, each level of α exposes a qualitatively distinct subset of facial dynamics. The multiple accumulation images thus constitutes a complementary, multi-level description of the micro expression rather than a redundant set, thus providing a robust and comprehensive input representation for the downstream recognition network. In this paper, we define the set of magnification intensities α ∈ {1, 5, 9, 13, 17, 21, 25, 29}. Here, α= 1 serves as the baseline, representing the raw input sequence where no motion magnification is applied. The upper bound is strategically capped at α = 29 to balance the amplification of subtle facial motion signals against the introduction of magnification artifacts. According to the fundamental principles of EVM, excessive amplification could lead to significant ringing artifacts and the non-linear boosting of high-frequency noise, which may obscure the underlying facial morphology [20]. Thus, α = 29 represents a practical threshold where subtle facial motion is emphasized before the signal-to-noise ratio (SNR) severely degrades. To validate this selection, we conducted comprehensive experiments across various combinations of α values. As shown in Table 1, the full integration of both low- and high-amplification regimes (from the baseline α = 1 to the high-intensity α = 29) consistently yielded the highest Accuracy, UAR, and UF1 scores on the CASME II and SMIC datasets.

### 3.2. Multi-Level Representation Based Classification Using a Lightweight Spatiotemporal Network

The set of the *N* accumulation images $\left\{f_{1},f_{2},\;\ldots ,f_{N}\right\}$ (for *N* = 8) constructed along the magnification intensity axis in Section 3.1 provides a rich and multi-level characterization of micro expressions. However, the optimal integration of this multi-level representation into a downstream classification network presents a non-trivial architectural challenge. A baseline approach would involve treating the accumulation images as *N* input channels for a single multi-channel 2D CNN. While this enables parameter sharing and ensures a lightweight profile, forcing a single set of kernels to process the entire range of α could not be optimal. The facial motion signals inherent in magnification regimes of different intensity levels are qualitatively distinct: lower α levels yield sparse, high-fidelity activations localized to primary muscle actions, whereas higher α levels reveal spatially diffused secondary deformations prone to saturation. To prevent the dilution of these discriminative features, we partition the eight accumulation images into two distinct groups: a low-amplification stream ($f_{1},\ldots ,f_{4}$ for α ∈ {1, 5, 9, 13}) and a high-amplification stream ($f_{5},\ldots ,f_{8}$ for α ∈ {17, 21, 25, 29}). By employing a dual-branch architecture where each branch utilizes its own shared-kernel multi-channel CNN, the model preserves the computational efficiency of kernel sharing while allowing each branch to specialize in the unique statistical characteristics of its respective amplification regime.

Beyond spatial appearance, the representation $\left\{f_{1},f_{2},\;\ldots ,f_{8}\right\}$ is able to encode critical dynamics along the magnification intensity axis. The evolution of facial motion evidence as α increases (specifically, the sequence in which regions emerge and the transitions between contiguous magnification levels) serves as a potent discriminative cue. To capture these dynamics, we introduce a TM [25] realized as a lightweight 1D-convolutional layer that slides along the magnification intensity axis to fuse adjacent α levels. Given its 1D kernel operating on single channel maps, the TM introduces negligible computational overhead. To further optimize the architecture, we place a temporal mixer before the network branches out. The eight accumulation images are stacked into a single sequence along the magnification intensity axis and jointly processed by the STM, after which the mixed sequence is partitioned into its respective low- and high-amplification halves for Branches A and B. This design achieves two primary objectives: (1) *Parameter Efficiency:* Sharing the TM across the entire magnification spectrum significantly reduces the total weight count compared to instantiating separate mixers for each branch. (2) *Cross-Regime Continuity:* It eliminates the artificial discontinuity at the boundary between the amplification groups. Since the transition from α = 13 to α = 17 represents a smooth progression on the magnification intensity axis, the STM enables the network to model the continuous evolution of motion-magnified facial dynamics. This ensures that cross-regime interactions are systematically preserved, thereby providing the downstream classifier with a temporally coherent feature space. It is worth noting that the STM used in the proposed method operates differently from the conventional LSTM which has been widely adopted in expression recognition. Immediately following the multi-level motion magnification stage, the STM with a 1D kernel observes only a few adjacent frames at a time. Its primary role is to act as a micro-motion filter, making it well-suited for capturing instantaneous fractional displacements within a short sequence (*N* = 8) of motion-magnified faces with increasing magnification intensities. In contrast, the LSTM operates at a higher abstraction level to model global sequence characteristics by taking the aggregated feature vectors from the entire video sequence as its input. Thus, LSTM could be useful for tracking how the motions evolve, sustain, and decay over the full duration of the expression.

Given the limited volumes of publicly available MER benchmarks, such as CASME II and SMIC, conventional deep and wide architectures often suffer from overfitting. To address this limitation, the proposed method adopts a shallow CNN backbone for each branch, maintaining an identical topology with independent weights to act as an implicit regularizer. To compensate for the reduced depth without compromising discriminative power, we integrate a CBAM before the final flattening layer. The dual-attention mechanism of CBAM is uniquely suited to the sparse and localized nature of micro expressions: (1) *Channel Attention (Adaptive Feature Selection):* This adaptively re-weights feature channels to emphasize filters most responsive to subtle motion patterns while attenuating those dominated by irrelevant identity-related or background appearance. (2) *Spatial Attention (Region-Specific Localization):* This generates a 2D mask to focus the network on facial sub-regions where motion occurs, such as the brow or lip corners, while effectively suppressing noise in static skin areas. Operating on semantically meaningful feature maps, CBAM acts as a data-driven analogue of an AUs [28,35] selector, achieving precise localization without explicit landmark supervision. This synergy between a shallow backbone and CBAM ensures high structural efficiency; the former minimizes parameter counts to prevent overfitting on small datasets, while the latter recovers the spatial selectivity typically associated with deeper networks.

**Figure 5:**
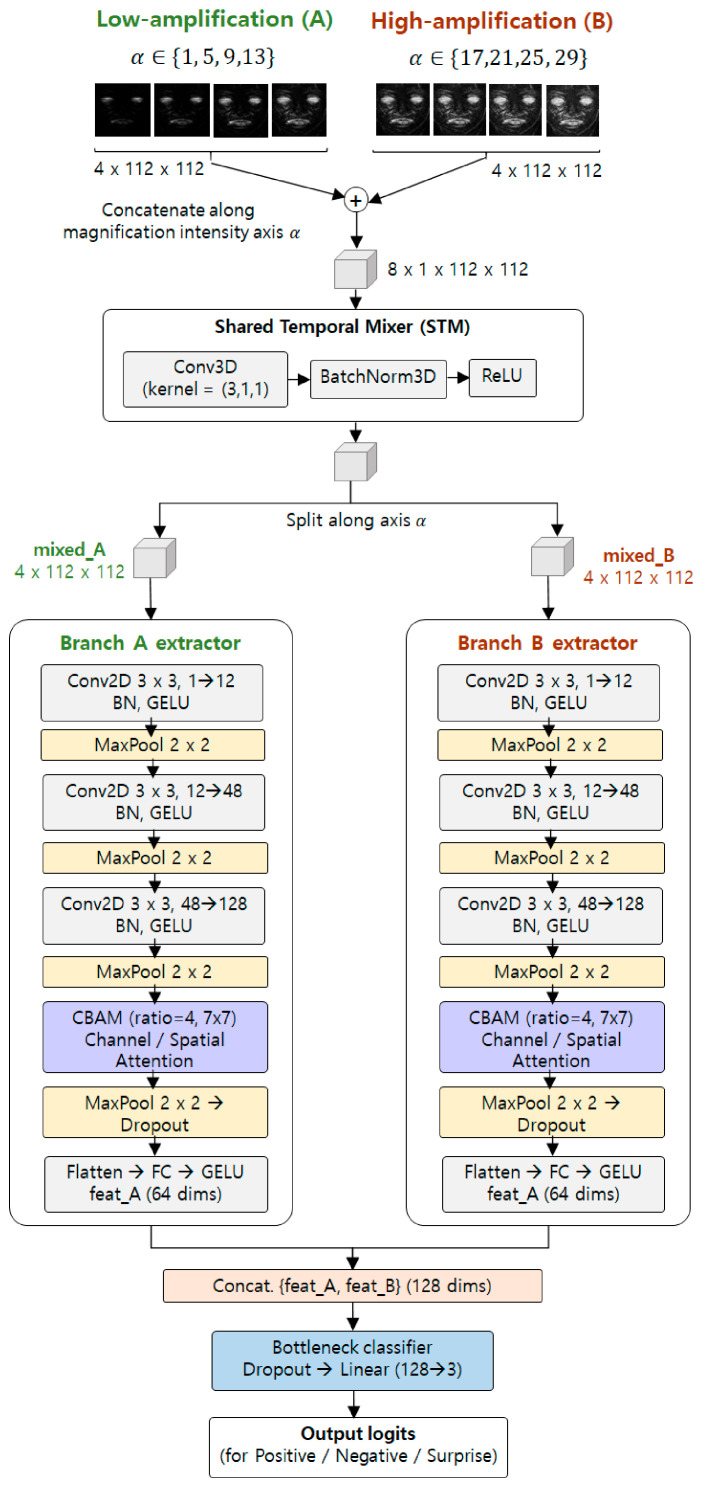
Detailed architecture of the proposed method.

Figure 5 summarizes the overall architecture of the proposed network. The data flow begins with two 4-frame sequences, each with a shape of (4, 112, 112), representing the low- and high-amplification accumulation images respectively. These sequences are concatenated along the magnification intensity axis into a single tensor of shape (8, 1, 112, 112) and fed into the STM. The STM is implemented as a single Conv3D layer with a kernel size of (3, 1, 1), padding (1, 0, 0), and a single input/output channel. This configuration acts as a 1D sliding window that fuses every magnification level with its adjacent neighbors along the α axis. To ensure stable gradient flow and minimal overhead, the STM includes a BatchNorm3D layer [36], a ReLU activation [37], and a residual skip connection. Remarkably, the STM adds only three convolutional weights and two BatchNorm parameters, making its impact on the total parameter budget practically negligible. Post-mixing, the tensor is split back into mixed_A and mixed_B (each of shape (4, 112, 112)) and fed into their respective branches. Each branch employs an identical shallow CNN: *(1) Feature Extraction:* Three Conv2D stages (1 → 12 → 48 → 128) with 3 × 3 kernels, BatchNorm, and GELU activations [38]. *(2) Spatial Reduction:* 2 × 2 max-pooling follows each stage, reducing the spatial resolution from 112 × 112 to 14 × 14. *(3) Attention and Dropout:* A CBAM block (reduction ratio 4, 7 × 7 spatial kernel) is applied to the final 128 channel feature maps, followed by a 2 × 2 max-pooling and a Dropout2D [39]. The resulting maps are flattened and projected into 64-dimensional feature vectors (feat_A, feat_B ∈ $R^{64}$) through a fully connected layer with GELU non-linearity. These vectors are concatenated into a unified 128-dimensional representation. Finally, a bottleneck classifier produces the logits for the target emotion classes (e.g., Positive, Negative, Surprise). To determine the final emotion class without introducing any arbitrary decision thresholds, the network employs the standard argmax function, which selects the emotion class index corresponding to the highest logit value.

## 4. Experimental Results

In this section, we presented a comprehensive evaluation of the proposed method using two widely used MER benchmark datasets: CASME II and SMIC. The characteristics of the benchmark datasets used in our experiments were summarized as follows:

(1) CASME II: developed by the Chinese Academy of Sciences, this dataset contained spontaneous micro expressions captured at 200 fps with a high spatial resolution (approx. 280 × 340 pixels for the facial region). It comprised 255 sequences from 26 subjects, featuring detailed annotations for emotion categories, AUs, and onset-apex-offset frames.
(2) SMIC: this dataset provides samples elicited from 16 participants. We specifically utilized the SMIC-HS (High-Speed) subset, recorded at 100 fps, which contained 164 clips. These samples were categorized into three broader emotion classes: Positive (51 samples (=sequences)), Negative (70), and Surprise (43) [12,40].

To ensure a rigorous and fair comparison with state-of-the-art methods, we adopted the LOSO cross-validation protocol [3]. In this protocol, the dataset contains micro expression samples collected from multiple distinct human subjects, and the validation was executed through an iterative process where each subject took a turn as the evaluation target. The exact partitioning mechanism operates as follows: (1) *Subject-Based Separation:* In each evaluation fold, all micro expression sequences belonging to one specific subject were completely set aside and allocated solely as the test set. (2) *Independent Training Pool:* Concurrently, the video clips from all the remaining subjects were combined to form the training set. This meant the model was trained on a group of individuals that did not include the test person. This process ensured a strict boundary between training and testing. (3) *Complete Rotation:* This entire procedure was repeated iteratively until every single subject in the dataset had been used as the test set exactly once. The final accuracy metrics were calculated by combining the results from all these independent rounds. Through this strict subject-level isolation, we eliminated any possibility of identity bias or data leakage, ensuring that the model was evaluated solely on its ability to generalize to completely unseen individuals.

### 4.1. Comparative Evaluation on Recognition Accuracy

To ensure consistency and allow for a rigorous comparison with state-of-the-art methods, we followed the emotion mapping protocol as in [12,40]. In the case of CASME II, which originally contained five emotion categories, the samples were remapped into three common classes: Positive, Negative, and Surprise. Under this protocol, the Positive class consists of 32 samples, the Negative class comprised 88 samples (formed by merging the Disgust and Repression categories), and the Surprise class included 25 samples. In the case of the SMIC dataset, no additional mapping was required as the data was natively categorized into the three emotion classes. The distribution for SMIC consisted of 51 Positive samples, 70 Negative samples, and 43 Surprise samples. Table 1 summarizes the attributes of the datasets used in this experiment.

**Figure 6:**
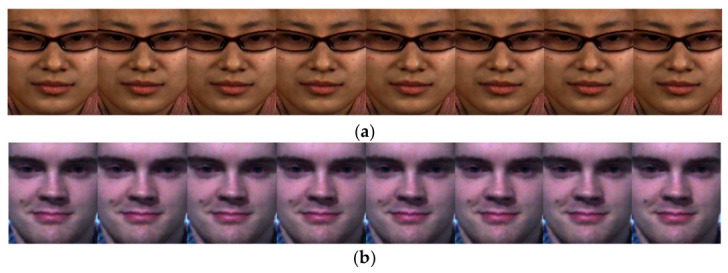
Examples of cropped face images. (**a**) CASME II dataset. (**b**) SMIC dataset.

To ensure the reproducibility and efficiency of the proposed model, all input facial images were resized to a standard resolution of 112 × 112 pixels. These cropped facial regions (Figure 6) were obtained directly from the preprocessed versions provided by the original dataset authors, ensuring that the input data maintained high fidelity and consistency across the CASME II and SMIC benchmarks. The neural network was trained for a total of 110 epochs using the Adam optimizer, which was selected for its robust performance in handling sparse motion features. The initial learning rate was set to 0.001. To facilitate stable convergence and prevent overfitting during the later stages of training, a learning rate decay strategy was implemented; specifically, the learning rate was reduced by 50% after the 50-th epoch. This adaptive scheduling allowed the model to effectively fine-tune its parameters once the global features had been sufficiently captured. To evaluate the performance of the proposed method, we employed three standard metrics: Accuracy, Unweighted Average Recall (UAR), and Unweighted F1-score (UF1). These metrics were selected to provide a comprehensive assessment, particularly given the inherent class imbalance present in spontaneous MER datasets. Accuracy served as the most intuitive measure, representing the ratio of correctly predicted samples to the total number of samples across all classes. It was calculated as follows:

(1) $$Accuracy=\frac{\left(TP+TN\right)}{\left(TP+TN+FP+FN\right)}.$$

However, since accuracy could be misleading when certain emotion classes were more prevalent than others, we utilized Unweighted Average Recall (UAR). UAR calculated the recall (sensitivity) for each individual class and took their arithmetic mean, ensuring that the model’s ability to recognize minority classes was treated with equal importance regardless of the sample size. The formula was expressed as:

(2) $$\mathrm{UAR}\;=\;\frac{1}{C}\sum_{i=1}^{C}\frac{{TP}_{i}}{S_{i}},$$

where *C* is the number of classes and *S_i_* is the total number of samples in the *i*-th class. Finally, we reported the Unweighted F1-score (UF1), which was the standard for evaluating imbalanced MER benchmarks. UF1 calculated the F1-score for each class (i.e., the harmonic mean of precision and recall) and averaged them. This metric provided a balanced view of the model’s robustness by accounting for both false positives (FPs) and false negatives (FNs) across all categories:

(3) $${F1}_{i}=\frac{2\times {Precision}_{i}\times {Recall}_{i}}{{Precision}_{i}+{Recall}_{i}},\;UF1=\;\frac{1}{C}\sum_{i=1}^{C}{F1}_{i}.$$

By reporting these three metrics, we ensured that the performance of the proposed method was validated not just by its overall hit rate, but also by its consistency and reliability in identifying subtle emotional shifts across diverse and unbalanced data categories.

We conducted an experiment to investigate the impact of various magnification intensities on recognition performance and how the integration of low- and high-amplification regimes provided complementary cues for micro expression analysis. The experimental results were shown in Table 2. A comparative analysis revealed that the multi-level integration encompassing both low-amplification (α ∈ {1, 5, 9, 13}) and high-amplification (α ∈ {17, 21, 25, 29}) regimes consistently outperformed any single-regime subsets across all evaluated metrics. Furthermore, on the CASME II dataset, the proposed dual-branch approach utilizing the full spectrum of intensities achieved a significant accuracy of 79.31% and a UF1 score of 75.33%, marking a substantial breakthrough over the restricted-regime baselines. These results validated the hypothesis that low-intensity magnification and high-intensity magnification could provide mutually complementary motion cues. Further, this demonstrated that our dual-branch architecture effectively integrated the multi-level representation to achieve superior recognition performance compared to single-branch architecture.

**Figure 7:**
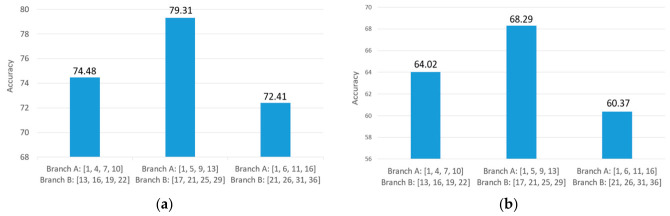
Performance comparison across three different ranges of magnification intensities α in dual-branch architecture of the proposed method. (**a**) CASME II dataset. (**b**) SMIC dataset.

As shown by Liu et al. [41], the magnification intensity α = 40 severely distorted facial images and degraded recognition accuracy, while α = 30 began to clearly exhibit visible noise artifacts. This was consistent with the theoretical limitation of linear EVM identified by Wu et al. [20], where large α values violated the first-order Taylor approximation and caused intensity clipping. Accordingly, we restricted our magnification intensity range to α ∈ {1, 5, 9, 13, …, 29} to balance motion enhancement with artifact suppression. To evaluate this threshold empirically, we conducted an ablation analysis by adjusting the maximum magnification intensity (max(α)). As shown in Figure 7, the results showed a general tendency where the accuracy peaked at max(α) = 29. Setting the upper limit lower (max(α) = 22) led to a slight decrease in accuracy, likely due to insufficient motion enhancement of the subtlest dynamics. Conversely, when the interval between motion magnification intensities was increased from 4 to 5, the max(α) consequently shifted upward to include higher values such as α = 31 and α = 36. Under this configuration, we observed a sharp performance degradation, with the accuracy dropping abruptly by approximately 7% to 8%. This empirical drop aligned with the expectation that over-amplification introduced unwanted artifacts and high-frequency noise. These results indicated that max(α) = 29 served as a practical, acceptable upper bound for balancing motion visibility and artifact suppression.

An additional experiment was conducted to investigate the individual and combined contributions of the STM and CBAM modules to the overall recognition performance. By evaluating the performance variations according to the presence or absence of each module within the baseline shallow CNN architecture, we aimed to explore how temporal dynamics and spatial attention mechanisms enhanced the discriminative power of the model. The experimental results in Table 3 revealed that the synergistic integration of both modules led to the most significant performance gains compared to using each module in isolation. On the CASME II dataset, the complete model incorporating both STM and CBAM achieved an accuracy of 79.31% and a UF1 of 75.33%, marking an improvement of over 17% compared to the baseline configuration without these components. This consistent performance tendency across both the datasets demonstrated that the STM effectively blended subtle temporal features while the CBAM emphasized crucial spatial information. Consequently, this ablation study validated that each proposed component played a vital and complementary role in achieving robust and high-performance micro expression recognition.

**Figure 8:**
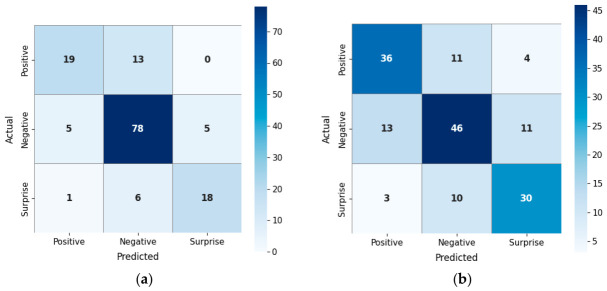
Confusion matrices of proposed method. (**a**) CASME II dataset. (**b**) SMIC dataset.

The confusion matrices in Figure 8 highlighted the performance of the proposed method on the CASME II and SMIC datasets. In both cases, the strong diagonal dominance indicated that the model effectively captured the discriminative features required for accurate emotion classification. This consistent performance tendency demonstrated the robustness and generalizability of the proposed method in effectively distinguishing between Positive, Negative, and Surprise categories under varying experimental conditions. Specifically, while the model exhibited an exceptionally high recognition rate for the Negative class in the CASME II dataset, it maintained a remarkably balanced classification performance across all three emotion categories in the SMIC dataset despite the inherent complexity of the samples. Overall, these results showed that the proposed architecture minimized inter-class interference and provided a highly reliable mapping of subtle facial motion dynamics to their respective emotional labels.

To provide a comprehensive evaluation of the proposed method, we compared its performance against various MER methods, which could be broadly categorized into five groups based on their feature extraction strategies and architectural designs (refer to Table 4). The first category consisted of hand-crafted feature-based methods, such as LBP-SIP [31] and LBP-TOP [8], which relied on manually designed descriptors to capture subtle skin texture changes and local facial variations. While these methods established the foundation for MER, they often struggled with the non-linear dynamics of spontaneous expressions compared to deep learning approaches. The second category, full sequence analysis with spatiotemporal analysis, included models such as ELRCN [34] and 3D CNN [42]. These methods focused on modeling the entire temporal flow of a micro expression rather than relying on a single representative frame. By processing the complete video sequence, they attempted to capture the continuous evolution of facial muscle movements, although this often resulted in increased computational complexity and higher memory requirements. The third category consisted of Vision Transformer (ViT)-based approaches, such as DeiT [11] and Light ViT [12]. These methods leveraged self-attention mechanisms to capture global context and long-range dependencies within facial images. While ViT-based models demonstrated high representative power, they were often characterized by a massive number of parameters which posed significant challenges for deployment on resource-constrained hardware. The fourth category was the dual-stream analysis which comprised architectures that utilized parallel branches to extract complementary information, such as spatial and temporal features or different emotional cues. Methods like DSSN [13], GEME [14], DSTNet [15], and dual-branch attention network (called Dual-ATME) [16] fell into this group, aiming to maximize recognition accuracy through multi-modal or multi-branch integration. We also compared against state-of-the-art methods with advanced geometric and semantic approaches. This included a graph structure representation based approach (Vision GNN [17]), AU-detection based approach (AU-guided data augmentation [18]), and fine-grained dynamic analysis (fine-grained 3D facial reconstruction [19]).

**Table 4:** Comparisons with other MER methods. (**a**) CASME II and (**b**) SMIC datasets.

**(a)**				
Category	Method	CASME II		
		Acc.	UAR	UF1
Hand-crafted	LBP-TOP (2011) [8]	-	54.29	50.26
	LBP-SIP (2014) [31]	-	52.81	53.69
Spatiotemporal analysis/ 3D CNN	CBAMNet (2020) [40]	69.92	-	-
	ELRCN (2018) [34]	-	43.96	50.00
Vis. Trans.	DeiT (2021) [11]	-	68.14	69.94
	Light ViT (2022) [12]	-	69.97	72.51
	Light ViT without transfer learning (2022) [12]	-	63.48	65.24
Dual-stream	DSTNet (2025) [15]	73.98	-	74.24
	Dual-ATME (2023) [16]	81.70	75.10	76.50
Advanced geometric & semantic approaches	Vision-GNN (2025) [17]	-	71.95	71.29
	AU-guided data augmentation (2026) [18]	73.60	68.90	-
	Fine-grained 3D facial reconstruction (2026) [19]	53.75	-	-
	Proposed	79.31	73.44	75.33
**(b)**				
Category	Method	SMIC		
		Acc.	UAR	UF1
Hand-crafted	LBP-TOP (2011) [8]	-	52.80	20.00
	LBP-SIP (2014) [31]	-	51.42	44.52
Spatiotemporal analysis/ 3D CNN	CBAMNet (2020) [40]	54.84	-	-
	3D CNN (2019) [42]	55.49	-	-
Vis. Trans.	DeiT (2021) [11]	-	68.81	69.7
	Light ViT (2022) [12]	-	73.56	71.41
	Light ViT without transfer learning (2022) [12]	-	60.18	62.15
Dual-stream	DSSN (2019) [13]	63.41	**-**	64.62
	GEME (2020) [14]	64.63	60.23	61.58
	DSTNet (2025) [15]	71.34	-	72.45
	Dual-ATME (2023) [16]	64.60	65.80	64.60
Advanced geometric & semantic approaches	Vision-GNN (2025) [17]	-	64.00	64.35
	AU-guided data augmentation (2026) [18]	51.20	46.60	-
	Proposed	68.29	68.69	68.41

As shown in Table 4, the proposed method showed superior performance compared to several methods. The comparative analysis of the results was summarized as follows: First, a substantial performance gap was observed when comparing the proposed method to hand-crafted feature-based methods. Traditional descriptors such as LBP-SIP and LBP-TOP recorded accuracies below 55% on both the datasets, failing to capture the complex non-linear dynamics of spontaneous micro expressions. Second, while full sequence approaches such as CBAMNet and 3D CNN utilized 3D convolutions to model temporal dynamics, they achieved significantly lower results than the proposed method. Specifically, on the CASME II dataset, CBAMNet reached an accuracy of 69.92%, which was approximately 10% lower than our result of 79.31%. This suggested that processing long sequences (e.g., 16 frames) could introduce redundant temporal information or suffer from overfitting on small-scale datasets. Third, the proposed method demonstrated highly competitive performance when compared to recent Vision Transformer and dual-stream architectures, such as Light ViT and DSTNet. On the CASME II, our proposed method outperformed these models, which was a notable improvement over the 69.97% (UAR) of Light ViT and 73.98% (Accuracy) of DSTNet. While Light ViT and DSTNet demonstrated impressive recognition accuracy that was comparable to or occasionally higher than our results on specific benchmarks (refer to the results on the SMIC dataset in Table 4b), a closer examination revealed significant limitations regarding their suitability for practical, real-time applications. Although Light ViT achieved competitive performance by integrating convolution layers with Vision Transformers, it required approximately 5.6 M parameters and relied on extensive grid searches to determine optimal hyper parameters, which complicated its deployment in resource-constrained environments [12]. Furthermore, its overall architectural complexity remained a hurdle for integration into low-power embedded systems. The limitations were even more pronounced in the case of DSTNet. Despite its high accuracy, this model did not achieve a true end-to-end training or inference pipeline because it required external preprocessing through additional networks for optical flow [15]. This dependency on heavy preprocessing modules significantly increased the computational burden and latency. Dual-ATME achieved higher accuracy on CASME II (81.70%) than the proposed method (79.31%), while the proposed method outperformed it on SMIC (68.29% vs. 64.60%). The performance gap on CASME II was largely attributed to the high-quality temporal annotations available in that dataset [27]. Dual-ATME relied on onset and apex frame pairs to compute optical flow features, and CASME II’s 200 fps capture rate and precise per-sample annotations provided near-ideal conditions for this apex-dependent design. The HARS module [16] further reinforced performance by focusing on the eyebrow and mouth ROIs, which were the most discriminative regions under that dataset’s AU distribution. However, in the SMIC dataset, the lower frame rates of 100 fps could lead to less precise apex localization. Consequently, the apex dependency of Dual-ATME resulted in degraded recognition performance. On the other hand, the proposed apex-free framework, by design, avoided this bottleneck. Although the newly emerging advanced geometric and semantic approaches showed overall competitive performance, they commonly suffered from structural limitations in practical deployment. Methods utilizing graph structures [17] or fine-grained 3D facial reconstruction [19] frequently relied on complex optimization processes or required external pre-trained models to estimate pseudo-ground-truth labels, which introduced potential alignment errors and optimization bottlenecks. Furthermore, the framework in [18] strictly relied on external, pre-trained AU detection modules to guide the data augmentation, making the overall pipeline highly dependent on the accuracy of third-party models. In contrast, the proposed method could achieve a superior balance by maintaining a highly compact architecture and providing a streamlined end-to-end inference capability, as the amplification process did not require additional learnable parameters from external networks.

**Figure 9:**
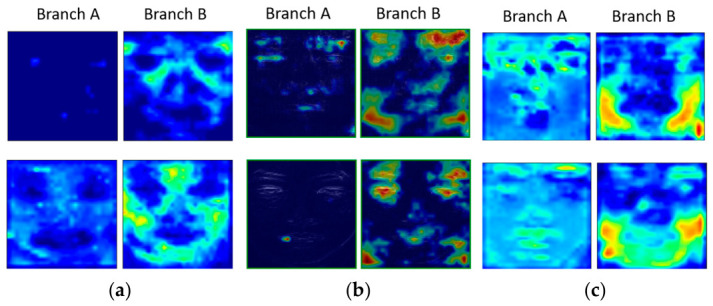
Comparison of Grad-CAM visualizations between a pair of test samples (i.e., input micro expression sequence) of a same emotion class from different human subjects. The six test samples used in this figure are correctly classified via the proposed method under the LOSO protocol. (**a**) Positive. (**b**) Negative. (**c**) Surprise.

To qualitatively analyze the architecture of the proposed method, Figure 9 illustrates the Gradient-weighted Class Activation Mapping (Grad-CAM) visualizations [43] for test samples under the LOSO protocol. The activation heatmaps suggested that the network could extract discriminative features depending on the emotion class. Distinct topological patterns were observed between different emotions (i.e., Positive, Negative, and Surprise). Within the same emotion class, the model showed a tendency to form relatively consistent attention patterns, even when processing different subjects with inherent identity discrepancies.

### 4.2. Further Analysis on Model Complexity

The computational complexity of the proposed model and all baseline architectures were quantified utilizing the FlopCountAnalysis utility from the fvcore library [44], which was a widely recognized standard for profiling FLOPs in computer vision research (e.g., used in Detectron2 [45] and PyTorchVideo [46]). All measurements were conducted in inference mode (model.eval()) with a batch size of 1. We reported multiply–accumulate operations (MACs) as the primary metric for computational efficiency, adopting the standard convention that 1 MAC was equivalent to 2 FLOPs [47]. Total parameter counts were determined using the parameter_count utility, encompassing all learnable parameters, including those within normalization layers. Consistent with the fvcore profiling convention, the reported FLOPs excluded element-wise operations—such as activations (ReLU, GELU), batch normalization (fused at inference), max-pooling, and element-wise multiplications in attention masks—given their negligible contribution relative to the heavy arithmetic load of convolution and matrix-multiplication operations. As summarized in Table 5, the proposed model comprised approximately 0.94 M parameters (942,770) and requires only 131 MMACs (262 MFLOPs).

We further evaluated the parameter efficiency of our method by comparing it against various baseline architectures and state-of-the-art MER models. As summarized in Table 6, the proposed method significantly reduced computational overhead compared to standard deep learning backbones. First, the difference in scale was highly evident when compared to traditional high-capacity networks. Our model, with only 0.94 M parameters, was approximately 12.4× lighter than ResNet18 (11.7 M), 65× lighter than AlexNet (61.1 M), and 147× lighter than VGG16 (138.3 M). Even compared to MobileNet V2 (3.5 M), which was optimized for mobile efficiency, our method achieved a 3.7× reduction in parameters while maintaining competitive accuracy. Furthermore, the proposed method maintained its competitive edge when evaluated against recent specialized MER architectures that reported parameter counts. Specifically: *(1) Vision Transformer-based models:* Our method was significantly more compact than DeiT (55.0 M) and Light ViT (5.6 M), being approximately 58.5× and 6× lighter, respectively. *(2) Sequence-based spatiotemporal analysis and dual-stream models:* While DSSN [13] also pursued an extreme lightweight design with 0.97 M parameters, our method (0.94 M) was slightly more compact while achieving a substantial performance gain on the CASME II dataset. Other complex structures such as CNN-LSTM (4.62 M) [33] and ELRCN (219 M) [34] exhibited a much larger memory footprint, making them less suitable for practical, real-world deployment. Within the compared dual-stream categories, Dual-ATME [16] and DSTNet [15] served as key benchmarks for model efficiency analysis, as they demonstrated competitive or slightly higher recognition performance compared to the proposed method. Since Dual-ATME’s parameter count was not originally reported, we instantiated its public repository to estimate its size, which yielded approximately 4.8 M parameters, making it 5.1× heavier than our method. Although DSTNet required 3.15 M parameters, its practical computational burden was even higher due to its strict dependence on external optical flow preprocessing. In contrast, the proposed method achieved a much more compact parameter footprint while eliminating any complicated preprocessing dependencies.

## 5. Conclusions

In this paper, we propose a new MER method which captures a rich, multi-level representation of subtle facial dynamics in videos by introducing a novel magnification intensity axis. By collapsing multi-level Eulerian motion sequences into structured accumulation images, the proposed method captures a rich, multi-level representation of subtle facial dynamics. This representation is efficiently processed through a lightweight shared STM and a dual-branch CNN with integrated attention modules (CBAM), which precisely isolate emotional deformations from identity-related noise. The proposed method offers three distinct advantages over existing MER methods: (1) By introducing a novel magnification intensity axis, the proposed model structurally resolves the dependency on apex frames. This approach fundamentally bypasses the inherent limitations of conventional methods that require separate automated apex-spotting algorithms. (2) The architecture achieves an optimal balance between model complexity and recognition accuracy. It delivers state-of-the-art performance while maintaining an exceptionally compact footprint of only 0.94 M parameters. (3) Unlike some existing methods that rely on external preprocessing networks, our framework constructs *N* accumulation images from multi-level motion magnification as a fixed signal-processing step. This ensures a streamlined inference pipeline without adding any learnable parameters, strictly limiting the total parameter count.

The proposed method provides a foundational step toward the practical deployment of MER by demonstrating that high performance can be achieved within an architecture suitable for resource-constrained environments. Furthermore, by introducing the magnification intensity axis to capture a broad spectrum of facial motion signals, the model achieves improved robustness and generalization across diverse recording conditions and individual variations, establishing a highly efficient yet flexible framework for MER.

## Figures and Tables

**Table 1:** The attributes of the MER datasets used in this experiment.

	Datasets	CASME II	SMIC
Attribute			
Number of subjects		24	16
Frame Rate (fps)		200	100
Average duration (frames)		66	37
Sample Distribution	Positive	32	51
	Negative	88	70
	Surprise	25	43

**Table 2:** Performance comparison across different combinations of magnification intensities α.

Combination of Magnification Intensities	CASME II			SMIC		
	Acc.	UAR	UF1	Acc.	UAR	UF1
[1, 5, 9, 13]	66.21	61.95	61.96	60.37	61.01	60.61
[17, 21, 25, 29]	64.14	58.75	58.43	61.59	62.85	62.55
[5, 13, 21, 29]	66.21	61.95	62.13	63.41	65.00	63.85
[1, 5, 9, 13, 17, 21, 25, 29]	69.66	59.66	63.01	64.02	65.30	64.62
Branch A: [1, 5, 9, 13] Branch B: [17, 21, 25, 29]	79.31	73.44	75.33	68.29	68.69	68.41

**Table 3:** Impact of the TM and CBAM on the recognition performance.

Types of Modules Applied to the Shallow CNN	CASME II			SMIC		
	Acc.	UAR	UF1	Acc.	UAR	UF1
without STM and CBAM	62.07	58.14	58.07	48.17	51.67	47.95
with STM only	63.45	62.72	61.16	62.80	65.29	63.08
with CBAM only	66.90	63.95	63.69	55.49	56.36	55.43
with STM and CBAM	79.31	73.44	75.33	68.29	68.69	68.41

**Table 5:** Details on model complexity analysis of proposed method.

Module	Sub-Module	Output Shape	No. Parameters	MACs
STM	Conv3D (1 → 1, k = (3, 1, 1))	(1, 8, 112, 112)	3	301,056
	BatchNorm3D + ReLU + Residual	(1, 8, 112, 112)	2	0
Branch A	Conv2D (4 → 12, 3 × 3) + BN + GELU + MaxPool	(12, 56, 56)	468	5,419,008
	Conv2D (12 → 48, 3 × 3) + BN + GELU + MaxPool	(48, 28, 28)	5328	16,257,024
	Conv2D (48 → 128, 3 × 3) + BN + GELU + MaxPool	(128, 14, 14)	55,680	43,352,064
	CBAM (ratio = 4, k = 7) + MaxPool + Dropout	(128, 7, 7)	8241	17,796
	Flatten + Linear (6272 → 64) + GELU	(64,)	401,472	401,408
	Subtotal		471,189	65,447,300
Branch B	(Identical topology to Branch A)	(64,)	471,189	65,447,300
Classifier	Dropout + Linear (128 → 3)	(3,)	387	384
Total			942,770 (≈0.94 M)	131,196,040 (≈0.131 G)
Total FLOPs	(1 MAC = 2 FLOPs)			262 M

**Table 6:** Comparisons with other MER methods on the number of parameters.

Method	No. Parameters
ResNet18	11.7 M
VGG16	138.3 M
AlexNet	61.1 M
MobileNet V2	3.5 M
CNN-LSTM (2016) [33]	4.62 M *
ELRCN (2018) [34]	219 M *
DSSN (2019) [13]	0.97 M
DeiT (2021) [11]	55.0 M
Light ViT (2022) [12]	5.6 M
Dual-ATME (2023) [16]	4.8 M ^+^
DSTNet (2025) [15]	>3.15 M
Vision-GNN (2025) [17]	7.1 M
Proposed	0.94 M (942.770)

* No. of parameters were directly cited from [13]. ^+^ No. of parameter for Dual-ATME [16] was not stated in the original paper and was estimated from the authors’ released source code.

## Data Availability

No new data were created.
